# Spontaneous Rupture of the Uterus before Labour

**Published:** 1848-03

**Authors:** Thomas F. Brownbilli

**Affiliations:** Surgeon to the Salford Workhouse


*Spontaneous Rupture of the Uterus before labour.* By Thomas F. Brownbilli, Esq., Surgeon to the Salford Workhouse.—M. A. Glover, aged 28 years, was of rather short stature, well proportioned, and had a healthy appearance. She had been married about eight years. In ten months after her marriage, after an ordinary labour of about nine hours' duration, she gave birth to a full-grown female child, which lived about four months. Soon after labour, which I understand was quite natural, she was seized with convulsions, followed by delirium, &c., which, continuing for a week or ten days, subsequently resulted in an attack of puerperal mania, for which she was afterwards admitted into the Manchester Workhouse. Here she remained about two months, and, as no improvement had taken place, was then sent to the Lancaster Asylum, whence, having been confined seven or eight months, she was discharged cured, and from that until the present time, has enjoyed uninterrupted good health, having been separated from her husband most of the time since her last confinement. She again became pregnant, and was admitted into the Salford Workhouse on the 4th of November last, in order to lie in.

She stated that in the beginning of the seventh month of gestation, whilst hanging out some cloths, she received a fall, which shook her violently, but did not cause her, either then or afterwards, any particular pain. On the 20th of November, at 6 A. M., after having passed a restless night, with occasional slight uterine pains, she began to vomit. This was followed by several pretty strong pains, during one of which she experienced, (to use her own expression,) a severe crack in the back, with a feeling of something suddenly giving away in her inside, which was immediately followed by a discharge of liquor amnii from the vagina. The midwife, Mrs. Livsey, (an intelligent and experienced person,) was accordingly sent for, and was soon in attendance. She found, upon examination, the os uteri nearly closed, hard, and incapable of admitting the point of the finger; there was a slight discharge of a dark brown colour from the vagina; the patient had vomited the contents of the stomach, and the pains had altogether subsided. Under these circumstances, she left her and found, on her return at 3 P. M., that she had had no pain during her absence; the os uteri was lower down, and more yielding, though not in the least dilated, and a slight discharge of water, tinged with blood, escaped whilst making the examination. She had not slept, nor felt the motion of the child since. Soon after, the waters broke. A dose of castor oil was now ordered.

On visiting her the following evening, at the request of Mr. Roberts, the governor of the Workhouse, I found the oil had been rejected by the stomach, and the vomiting had continued more or less to the present time, the matter at first being of a greenish yellow, and afterwards of a chocolate colour; labour had not in the least progressed, the os uteri remaining as before, if anything, more contracted; had no pains; complained of being weak and poorly, and, although several opiates had at short intervals been administered, she had as yet not slept, and with a feeble pulse, her countenance now began to assume an anxious expression.

November 22nd. About 11 A. M., she began to doze for short periods, but this state soon gave way to extreme restlessness, almost incessantly requiring her position to be altered. She now complained of severe pain in the middle of her back, and her pulse was evidently sinking. Between one and two o'clock her breathing became laborious, her finger nails turned livid, a continued gasping followed, and in this state she died.

The body was inspected, twenty-four hours after death, in the presence of several medical friends, and Mr. Roberts, the Governor. The abdomen was found to contain a large quantity (about two pints) of dark-coloured, uncoagulated blood, probably diluted with a portion of the liquor amnii, and this being partially removed, the first object that presented itself, entirely excluded from the womb, and partially covered by the omentum and small intestines, was a full-grown male child, that had evidently been dead several days, the first stage of putrefaction having commenced. On partially removing the child, which lay with its left shoulder to the womb, a large rupture of this organ was observed, extending from the centre of the fundus posteriorly along its whole length as far as the os uteri, leaving only a narrow rim surrounding it, and through which the child had escaped into the cavity of the abdomen. The length of the opening was about seven inches, and the uterus, which seemed perfectly healthy, was contracted over the firmly adherent placenta.

In the above unfortunate case there are several points worthy of general notice, and which are of peculiar interest to the obstetrician. The patient was at the end of her calculation, and had a well-formed pelvis; the child was full-grown, of average size, also well formed, and there existed between the two no disparity which would prevent the one easily passing through the other, supposing the presentation to be natural. From the time she first began to complain, up to her death, there was not the slightest pressure downwards; the os uteri was not at all dilated, and firmly resisted the introduction of the finger point, which effectually prevented me ascertaining the presentation of the child, nor was there any particular point or bulging perceptible in any part surrounding the os uteri, by which I could recognize its position. The os uteri projected downward a little way into the vagina, and above it seemed to lead to an obliterated cervix. Notwithstanding this state of things, from from the total absence, since the first commencement, of anything like strong or bearing down labour pains, even at the time the waters escaped, it, never occurred to me that the uterus had probably ruptured, which fact I first discovered at the *post-mortem* examination.

The cause of the rupture is involved in much obscurity. She had not over-exerted herself, nor had she received any bodily injury since the time she fell, and the shake the fall occasioned was not followed by any soreness, or other inconvenience. Towards the end of gestation, she was often low spirited, and entertained a presentiment to which she often gave expression, that she should not survive the birth of her child. There was no softening of stricture in the uterus, nor any indications of previous inflammation. The surrounding soft parts were healthy; the usual predisposing and exciting causes were all absent. Even supposing there was mal-position, of the fœtus, that the wall of the uterus should be endangered from such trivial pains seems surprising. The case altogether is remarkable, and it presents an instance of the least, I believe, in frequency—viz., the longitudinal rupture of this organ extending from the centre of the fundus posteriorly in a straight direction, to within half an inch of the posterior centre of the os uteri.—*Prov. Med. and Surg. Journal.*

